# Assessment of Parents' Knowledge Regarding Pediatric Glucose-6-Phosphate Dehydrogenase Deficiency in Saudi Arabia

**DOI:** 10.7759/cureus.50664

**Published:** 2023-12-17

**Authors:** Sawsan M Al Blewi, Rawiyah A Alessa, Lena D Alzahrani, Omar M Kheder, Rand A Alissa, Lama S Alharbi, Layan F Alsanad, Ahmed S Almuzaini

**Affiliations:** 1 Pediatric Hematology Oncology/Faculty of Medicine, University of Tabuk, Tabuk, SAU; 2 College of Medicine, Faculty of Medicine, University of Tabuk, Tabuk, SAU; 3 College of Medicine, Taibah University, Madinah, SAU; 4 College of Medicine, Majmaah University, Majmaah City, SAU; 5 College of Medicine, Ibn Sina National College, Jeddah, SAU; 6 College of Medicine, King Saud University, Riyadh, SAU; 7 College of Medicine, Qassim University, Buraydah, SAU

**Keywords:** saudi arabia, parents’ awareness, children, parents’ knowledge, glucose-6-phosphate dehydrogenase (g6pd) deficiency

## Abstract

Introduction

Glucose-6-phosphate dehydrogenase deficiency (G6PD) is recognized as the most common enzyme disorder globally, impacting over 400 million individuals. The disease is highly prevalent in Saudi Arabia. This study aimed to assess parents' awareness of G6PD in Saudi Arabia and identify misconceptions for targeted educational interventions, aiming to enhance awareness and condition management.

Methods

A structured online questionnaire was used to gather information from July 18th, 2023, to August 1st, 2023. The survey targeted parents of Saudi children who resided in various regions across Saudi Arabia and collected a total of 531 responses. Data analysis involved descriptive statistics, chi-square tests, and probit regression. A significance level of *p*<0.05 was employed to interpret the results.

Results

A statistically significant associations were found among parents with Glucose-6-phosphate dehydrogenase deficiency-deficient children, including gender-related (odd ratio = 2.91, 99% CI: 1.986-4.301), awareness of the genetic link (odd ratio = 2.49, 99% CI: 1.701-3.639), specific medications (odd ratio =1.890, 99% CI: 1.262-2.853), loss of appetite (odd ratio= 0.629, 95% CI: 0.398-0.990), jaundice (odd ratio = 3.01, 99% CI: 1.877-4.983), increased fluid intake (odd ratio= 1.53, 95% CI: 1.091-2.139), receiving blood transfusions (odd ratio = 1.54, 95% CI: 1.101-2.157), seeking online information (odd ratio = 1.92, 99% CI: 1.250-2.940), and consulting healthcare professionals (odd ratio = 3.24, 99% CI: 2.065-5.107).

Conclusion

Regional disparities in glucose-6-phosphate dehydrogenase deficiency awareness among parents in Saudi Arabia are evident, with the central region demonstrating the highest level of awareness. Understanding glucose-6-phosphate dehydrogenase deficiency risk factors, medication triggers, and clinical symptoms plays a significant role in parental knowledge, emphasizing the need for region-specific education and awareness programs.

## Introduction

In 1932, Walter Christian and Otto Warburg identified glucose-6-phosphate dehydrogenase (G6PD) deficiency in yeast and red blood cells [1]. The first report of G6PD deficiency in Saudi Arabia dates back to more than three decades ago, with Gelpi documenting the presence of this disease in various villages in the Eastern Province in 1965 [2]. G6PD deficiency is an X-linked genetic disorder that primarily affects males; females typically do not develop severe hemolytic anemia [3].

G6PD deficiency is recognized as the most common enzyme disorder globally, impacting over 400 million individuals [4]. This condition often leads to neonatal jaundice, which can result in complications like kernicterus, cerebral palsy, or even death. Patients with G6PD deficiency can present with severe clinical symptoms, including acute hemolytic anemia, jaundice, and chronic non-spherocytic hemolytic anemia [4]. This disorder arises from a genetic deficiency in the red blood cell enzyme G6PD, responsible for generating nicotinamide adenine dinucleotide phosphate and protecting RBCs from oxidative damage [5].

Newborns with G6PD deficiency typically remain symptom-free until they encounter stressors or triggers, such as certain foods like fava beans, specific medications, or infections [6,7]. A crucial aspect of treatment involves avoiding these triggers, and in severe cases, blood transfusions may be necessary [8].

The prevalence of G6PD deficiency varies significantly across different populations; for example, it is less than 1% in Japan but can be as high as 70% in Kurdish Jewish communities [9-11]. A study in the USA indicated that G6PD deficiency is most commonly found in African, Asian, Mediterranean, and Middle Eastern populations [12]. Additionally, specific triggers for G6PD deficiency include certain foods, drugs, and infections [13]. In 2016, a study reported a G6PD prevalence of 8.9% among Egyptian neonates [14]. Additionally, the study found that 95.9% of mothers were unfamiliar with the term "G6PD deficiency," while approximately 24% had heard of "Fava bean anemia." Moreover, only 24% were aware that certain drugs could trigger G6PD [15].

The Kingdom of Saudi Arabia comprises five main regions: the central, northern, eastern, western, and southern regions. Numerous studies [16,17] have explored glucose-6-phosphate dehydrogenase (G6PD) deficiency anemia, with a particular focus on the Eastern region, which is documented as endemic for G6PD deficiency anemia. The Eastern Province of Saudi Arabia consistently exhibits the highest G6PD deficiency prevalence in both genders, per multiple studies [16,17]. These studies underscore a substantial gender disparity in pediatric cases and among newborns. Among 25,628 newborn ICU/IMC admissions, G6PD deficiency occurred at 18.8%, highlighting the condition's significance in Saudi Arabia, especially in the Eastern province. Implementing newborn G6PD screening can aid early identification and care, reducing complications like hemolytic anemia. However, there are a few studies showing the assessment of Saudi parents’ knowledge regarding pediatric G6PD deficiency. Previous research has primarily centered on hospital-based studies, especially among neonates [4] and children [18,19].

Further research is needed to fully understand G6PD deficiency's impact on the country, given existing data gaps. This study aimed to assess parental knowledge of G6PD across all five regions, aiming to gain insights into its implications and formulate targeted recommendations. The following section outlines the research methodology used in the study.

## Materials and methods

The present investigation used a descriptive cross-sectional study design, which involved collecting data using a structured online questionnaire from July 18th, 2023, to August 1st, 2023. Invitations to participate in the inquiry were extended to all eligible populations that were easily reachable, and the survey was conducted upon the approval of the Local Research Ethics Committee at the University of Tabuk with the number UT-301-143-2023, and the recruitment process focused on those who willingly signed the online informed consent document.

The survey targeted parents of Saudi children who resided in various regions across Saudi Arabia. The study had targeted a minimum sample size of 377, which was determined using Raosoft, but the sample size was increased to 531 to minimize the bias. An online sample size calculator [20]. Cluster random sampling was implemented across all regions of Saudi Arabia, and study participants were included based on their convenient accessibility and willingness to participate in the research endeavor.

A validated questionnaire, translated into Arabic, was utilized to assess the general awareness of G6PD, understanding of G6PD risk factors, recognition of the clinical presentation of G6PD, awareness of G6PD treatment and sources of information on G6PD (independent variables), having a child with G6PH (dependent variable), and region, age, marital status, and education level (demographic variables) among the parents in Saudi Arabia.

The data was assessed and cleaned for any potential errors or discrepancies. The R language, specifically version 4.3.1 [21] was the main analytical tool. The relevant descriptive statistics were computed and then summarized in terms of frequency, percentage, and mean values. The analysis of categorical variables, stratified by demographics, was conducted using the Chi-square test. The odds ratios, both unadjusted and adjusted, were calculated via probit regression to assess knowledge of G6PD. The baseline for interpreting statistical significance values was set at the 95% confidence level (p <0.005).

## Results

Descriptive statistics

The research was carried out across the five regions of the Kingdom of Saudi Arabia, as delineated in Table 1. The prevalence of G6PD in Saudi Arabia was documented at 9.4%. A significant portion of the study's participants (35.7%) fell within the age bracket of 18 to 30 years, while the smallest proportion (15.4%) consisted of individuals aged over 50 years. The majority of the study's participants held a bachelor's degree or higher (65.6%). In contrast, those with an intermediate level of education constituted the smallest group, accounting for only 6.9%.

**Table 1:** Demographic statistics Notes: n = frequency (count); % = percent

Variable	Characteristic	n (%)
Age	18-30 years	186 (35.7%)
	31-40 years	136 (26.1%)
	41-50 years	119 (22.8%)
	More than 50 years	80 (15.4%)
Region	Central Region	131(25.1%)
	Northern Region	81 (15.5%)
	Eastern Region	105 (20.2%)
	Western Region	105 (20.2%)
	Southern Region	99 (19%)
Education level	Intermediate & below	36 (6.9%)
	Secondary & Diploma	143 (27.4%)
	Bachelor & above	342 (65.6%)
G6PD diagnosis	No	472 (90.6%)
	Yes	49 (9.4%)

Multivariate statistics

Regarding the general awareness of G6PD, a statistically significant association was exclusively observed among parents who had children with G6PD and their belief that the inheritance of G6PD was linked to the sex of the child. This noteworthy association was statistically significant among residents from the Central Region, Eastern Region, and Western Region.

Inferential statistics

Measures of association were conducted employing Chi-squared statistics, as demonstrated in Table 2, to assess the relationships between the dependent and independent variables. In sum, all the measures exhibited statistically significant associations, thereby prompting the further examination of all constructs in the subsequent section through logistic regression analysis.

**Table 2:** Measures of association between the dependent and the independent variables

Independent	Item		No	Yes	p-value
Awareness	Heard of fava bean anemia	No	232 (44.5%)	6 (1.2%)	<0.001
		Yes	240 (46.1%)	43 (8.3%)	
	Blood disorder	No	196 (37.6%)	4 (0.8%)	<0.001
		Yes	276 (53%)	45 (8.6%)	
	Genetic disease	No	298 (57.2%)	11 (2.1%)	<0.001
		Yes	174 (33.4%)	38 (7.3%)	
	Parent as carriers	No	402 (77.2%)	36 (6.9%)	0.033
		Yes	70 (13.4%)	13 (2.5%)	
	Gender-related	No	416 (79.8%)	18 (3.5%)	<0.001
		Yes	56 (10.7%)	31 (6%)	
Risk factors	Knowledge of risk factors	No	414 (79.5%)	21 (4%)	<0.001
		Yes	58 (11.1%)	28 (5.4%)	
	Family history	No	275 (52.8%)	11 (2.1%)	<0.001
		Yes	197 (37.8%)	38 (7.3%)	
	Infection triggers	No	362 (69.3%)	26 (5%)	<0.001
		Yes	11 (21.3%)	23 (4.4%)	
	Some medications	No	344 (66%)	14 (2.7%)	<0.001
		Yes	128 (24.6%)	35 (6.7%)	
	Eating Fava beans	No	241 (46.3%)	5 (1%)	<0.001
		Yes	231 (44.3%)	44 (6.4%)	
Clinical presentation	Pallor as a symptom	No	264 (50.7%)	9 (1.7%)	<0.001
		Yes	208 (39.9%)	40 (7.7%)	
	Loss of appetite	No	300 (57.6%)	22 (4.2%)	0.01
		Yes	172 (33%)	27 (5.2%)	
	Dizziness/light-headedness	No	283 (54.3%)	15 (2.9%)	<0.001
		Yes	189 (36.3%)	34 (6.5%)	
	Shortness of breath	No	306 (58.7%)	19 (3.6%)	<0.001
		Yes	166 (31.9%)	30 (5.8%)	
	Jaundice	No	327 (62.8%)	8 (1.5%)	<0.001
		Yes	145 (27.8%)	41 (7.9%)	
	Urine color change	No	355 (68.1%)	21 (4%)	<0.001
		Yes	117 (22.5%)	28 (5.4%)	
Treatment	Increased fluid intake	No	332 (63.7%)	21 (4%)	<0.001
		Yes	140 (26.9%)	28 (5.4%)	
	Receive blood transfusion	No	333 (63.9%)	21 (4%)	<0.001
		Yes	139 (26.7%)	28 (5.4%)	
Information	Online information	No	375 (72%)	12 (2.3%)	<0.001
		Yes	97 (18.6%)	37 (7.1%)	
	Healthcare professional	No	430 (82.5%)	16 (3.1%)	<0.001
		Yes	42 (8.1%)	33 (6.3%)	
	Educational events/seminars	No	431 (82.7%)	27 (5.2%)	<0.001
		Yes	41 (7.9%)	22 (4.2%)	

In general, healthcare professionals emerged as the primary sources of G6PD information, constituting 252 individuals, or 48.4% of the respondents. They were followed by the internet, with 140 individuals (26.9%), while family or friends accounted for 59 individuals (11.3%), and media for 38 individuals (7.3%). Additionally, a smaller proportion cited other sources, totaling 32 individuals (6.1%).

**Figure 1:**
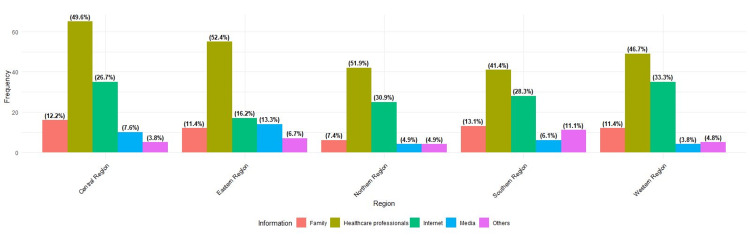
Distribution of sources of information on G6PD deficiency

Upon categorizing the sources of information on G6PD deficiency based on regional distribution, it became evident that most participants across regions identified healthcare professionals as their primary source of information, as depicted in Figure 1, closely followed by the internet.

**Figure 2:**
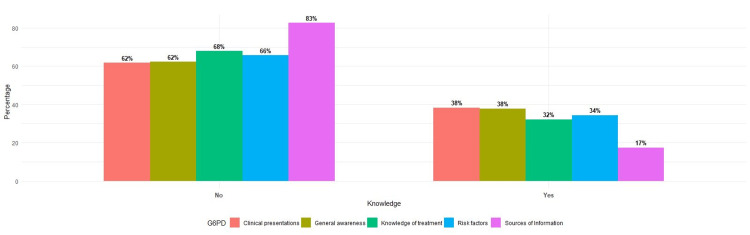
Knowledge of G6PD is based on the independent variable studied.

Figure 2 below illustrates the level of knowledge represented by the study population in the five regions of Saudi Arabia. It provides a comprehensive visual representation of the awareness and understanding of G6PD deficiency among parents in these regions, highlighting potential areas for targeted education and intervention.

Multivariate statistics

Regarding the general awareness of G6PD, a statistically significant association was exclusively observed among parents who had children with G6PD and their belief that the inheritance of G6PD was linked to the sex of the child. This noteworthy association was statistically significant among residents from the Central Region, Eastern Region, and Western Region, as indicated in Table 3.

**Table 3:** Awareness of G6PD deficiency Note:  *p<0.1; **p<0.05; ***p<0.01;  AdjOR: Adjusted odd ratio; CI: Confidence interval

	Dependent variable: Child with G6PD					
Independent variable	Overall	Central	Northern	Eastern	Western	Southern
Fava beans	1.607* (0.997-2.676)	-	-	-	-	-
Blood disorder	1.159 (0.671-2.062)	-	-	-	-	-
Genetic disease	1.435* (0.961-2.164)	-	-	-	2.805* (1.075-8.081)	-
Parents as carriers	0.914 (0.593-1.383)	-	-	-	2.059* (0.901-4.722)	-
Gender-related	2.911*** (1.986-4.301)	3.941*** (1.445-12.284)	-	2.999*** (1.560-5.890)	3.124** (0.087-0.349)	-
Constant	0.103*** (0.061-0.157)	-	-	-	-	-
Observations	521	131	81	105	105	99
Log Likelihood	-124.967	-16.482	-12.722	-37.511	-31.534	-6.167
Akaike Inf. Crit.	261.933	44.964	37.444	87.023	75.067	24.334

Knowledge of the risk factors associated with G6PD was assessed using five items, as outlined in Table 4. Among these items, only two were statistically significant in the overall model: parents who believed their knowledge made them susceptible to having children with G6PD and those who recognized that certain medications could lead to G6PD deficiency anemia. When considering the regional distribution, it is noteworthy that family history emerged as the sole significant factor in the Northern Region. In the Eastern Region, only awareness of medications as a risk factor was statistically significant, while in the Western Region, knowledge of the likelihood of having a baby with G6PD was the sole statistically significant factor. Conversely, neither the Central Region nor the Southern Region exhibited any statistically significant items in this regard.

**Table 4:** Risk awareness Note:  *p<0.1; **p<0.05; ***p<0.01; AdjOR: Adjusted odd ratio; CI: Confidence interval

	Dependent variable: Child with G6PD					
Independent variable	Overall	Central	Northern	Eastern	Western	Southern
Knowledge	2.485*** (1.701-3.639)	-	-	-	4.887*** (2.038-12.318)	-
Family history	1.184 (0.790-1.784)	-	0.254*** (0.062-0.813)	-	-	-
Infection triggers	0.954 (0.643-1.403)	-	-	-	-	-
Some medications	1.890*** (1.262-2.853)	-	-	3.273*** (1.633-6.894)	-	-
Eating fava beans	1.542* (0.957-2.557)	-	-	-	-	-
Constant	0.104*** (0.067-0.151)	-	-	-	-	-
Observations	521	131	81	105	105	99
Log Likelihood	-125.019	-16.209	-12.109	-34.838	-28.287	-8.709
Akaike Inf. Crit.	262.038	44.417	36.217	81.676	68.575	29.419

In assessing knowledge of clinical presentation, which was evaluated through six items, only two items, loss of appetite and jaundice, demonstrated statistical significance in the overall model, as indicated in Table 5. Regarding regional distribution, it's noteworthy that the Western Region was the only region where statistically significant items were observed within the clinical presentation construct. Specifically, the significant items were light-headedness and jaundice. In contrast, the Central Region, Northern Region, Eastern Region, and Southern Region did not yield statistically significant findings in this regard.

**Table 5:** Clinical presentation Note:  *p<0.1; **p<0.05; ***p<0.01; AdjOR: Adjusted odd ratio; CI: Confidence interval

Dependent variable: Child with G6PD						
Independent variable	Overall	Central	Northern	Eastern	Western	Southern
Pallor as a symptom	1.493 (0.915-2.448)	-	-	-	-	-
Loss of Appetite	0.629** (0.398-0.990)	-	-	0.455* (0.195-1.018)	-	-
Light-headedness	1.009 (0.609-1.663)	-	-	-	0.189** (0.044-0.720)	-
Shortness of breath	0.983 (0.636-1.519)	-	-	-	-	-
Jaundice	3.012*** (1.877-4.983)	-	-	2.774* (0.976-10.506)	4.180*** (1.581-12.106)	-
Urine color change	1.275 (0.854-1.908)	-	-	-	-	-
Constant	0.129*** (0.090-0.176)	-	-	-	-	-
Observations	521	131	81	105	105	99
Log Likelihood	-132.631	-19.651	-10.732	-37.827	-32.687	-8.718
Akaike Inf. Crit.	279.261	53.303	35.464	89.655	79.375	31.436

In examining knowledge of treatment, which was assessed using two items, all of them exhibited a statistically significant positive relationship with parents who had children with G6PD, as shown in Table 6. Regarding regional distribution, statistically significant relationships were observed in specific regions. In the Central Region, a significant relationship was identified among parents who believed that individuals with G6PD might require blood transfusions. Meanwhile, in the Western Region, a statistically significant relationship was found among parents who believed that individuals with G6PD deficiency needed to increase fluid intake during an attack. However, no statistically significant findings were reported from the Northern, Eastern, and Southern Regions.

**Table 6:** Treatment Note:  *p<0.1; **p<0.05; ***p<0.01; AdjOR: Adjusted odd ratio; CI: Confidence interval

Dependent variable: Child with G6PD						
Independent variable	Overall	Central	Northern	Eastern	Western	Southern
Increased fluid intake	1.528** (1.091-2.139)	-	-	-	2.685*** (1.357-5.405)	-
Receive blood transfusion	1.541** (1.101-2.157)	3.898*** (1.536-12.023)	-	-	-	-
Constant	0.188*** (0.148-0.236)	-	-	-	-	-
Observations	521	131	81	105	105	99
Log Likelihood	-152.151	-21.594	-17.364	-50.237	-38.337	-9.453
Akaike Inf. Crit.	310.303	49.188	40.728	106.474	82.674	24.907

The knowledge of sources of information on G6PD deficiency, which was assessed using three items (Table 7), exhibited statistically significant relationships in the overall model. Specifically, significant relationships were observed for obtaining information from online sources and from healthcare professionals. When considering regional distribution, the pattern of significance varied across regions. In the Central and Western Regions, obtaining information from healthcare professionals was statistically significant. In the Western Region, there was also a statistically significant relationship with the source of information through educational events or seminars. However, no significant relationships were reported among parents residing in the Northern, Eastern, and Southern Regions concerning these sources of information. 

**Table 7:** Information Note:  *p<0.1; **p<0.05; ***p<0.01; AdjOR: Adjusted odd ratio; CI: Confidence interval

Dependent variable: Child with G6PD						
Independent variable	Overall	Central	Northern	Eastern	Western	Southern
Online information	1.922*** (1.250-2.940)	-	-	1.952* (0.959-3.991)	-	-
Healthcare professional	3.237*** (2.065-5.107)	8.127*** (2.219-37.032)	-	2.068* (0.983-4.410)	4.726*** (1.743-13.308)	-
Educational seminars	1.252 (0.780-1.990)	-	-	-	2.713** (1.033-7.060)	-
Constant	0.136*** (0.102-0.175)	-	-	-	-	-
Observations	521	131	81	105	105	99
Log Likelihood	-114.554	-15.828	-6.236	-41.887	-32.687	-8.718
Akaike Inf. Crit.	237.108	39.656	20.472	91.773	79.375	31.436

Among individuals aged 31-40 years old (Table 8), a statistically significant relationship was identified with the source of information being healthcare professionals. This suggests that parents in this age group were more likely to acquire information about G6PD deficiency from healthcare professionals compared to other age groups. For parents with a secondary or diploma level of education, significant findings were observed in two areas. First, they demonstrated a noteworthy association regarding their knowledge of G6PD treatment, particularly understanding the role of increased fluid intake as a form of G6PD treatment. Second, parents with this level of education were more likely to obtain information about G6PD from healthcare professionals compared to those with different educational backgrounds. Regarding parents with a bachelor's degree and above, statistically significant relationships were noted in two domains. First, there was a significant association with clinical presentation, specifically the recognition of symptoms such as loss of appetite and jaundice. Additionally, parents with a bachelor's degree and above were more likely to obtain information about G6PD from healthcare professionals, signifying the importance of healthcare professionals as a primary source of information for this highly educated group.

**Table 8:** Significant items with demographic variables Note:  *p<0.1; **p<0.05; ***p<0.01; AdjOR: Adjusted odd ratio; CI: Confidence interval

		Dependent variable: Child with G6PD	
Independent variable	31-40 YEARS	Secondary & diploma	Bachelor & above
Some medications	-	-	1.860* (0.984-3.599)
Loss of Appetite	-	-	0.388*** (0.199-0.732)
Jaundice	-	-	5.235*** (2.391-14.931)
Increased fluid intake	-	2.442** (1.101-5.634)	-
Receive blood transfusion	-	-	-
Healthcare professional	2.683** (1.256-6.004)	3.372*** (1.675-6.967)	2.074** (1.199-3.673)
Constant	0.098*** (0.044-0.184)	0.124*** (0.065-0.210)	0.059*** (0.022-0.114)
Observations	136	143	342
Log Likelihood	-35.136	-35.268	-55.088
Akaike Inf. Crit.	84.272	84.537	124.177

## Discussion

This study assesses parents' knowledge regarding pediatric glucose-6-phosphate dehydrogenase deficiency in Saudi Arabia by focusing on knowledge at five different levels: general awareness, risk factors, clinical presentation, treatment, and sources of information. The study sampled parents from all five regions of the Kingdom of Saudi Arabia, namely the Central, Northern, Eastern, Western, and Southern regions. The central (Riyadh), western (Jeddah, Mecca), and eastern (Dammam) regions have lower poverty rates, reflecting educated working-class residents due to economic activities. Conversely, the northern region (Tabuk, Hail) and southern region (Abha, Jizan) face limited economic activity, likely contributing to lower education levels and potential G6PD knowledge disparities. In Saudi Arabia, cross-sectional studies have found G6PD deficiency prevalence rates ranging from 4.76% to 30.6% among newborns screened in Alhasa and AlQatif, emphasizing the vital role of parents in being educated about early signs for early detection and risk reduction [17,22,23]. In the current study, the average levels of awareness of G6PD deficiency were 37.8%, knowledge of G6PD risk factors was 34.5%, understanding of G6PD clinical presentation stood at 38.2%, knowledge about G6PD treatment reached 32.15%, and familiarity with sources of G6PD information was 17.4%. These low levels of awareness can be compared to those reported in a 2017 cross-sectional study in Saudi Arabia, which was conducted in three governmental hospitals in Riyadh city (Al-Yamama Hospital, Armed Forces Hospital, and King Salman Hospital). The study found that participants had poor knowledge and low awareness of the causes and prevalence factors of G6PD deficiency [22].

The general awareness of G6PD had an adjusted odds ratio (adjOR) of 2.9, signifying that parents who were informed about the connection between G6PD inheritance and a child's sex were approximately 2.9 times more likely to have a child with G6PD deficiency. This underscores the significant association between parental knowledge of this genetic link and the likelihood of having an affected child. The independent variable, "Knowledge that G6PD inheritance is linked to a child's sex," aligns with the findings reported in Alqahtani et al.'s [8] study, highlighting the crucial role of parental awareness in recognizing and seeking appropriate care or genetic counseling for G6PD deficiency in their child. The level of awareness of G6PD deficiency in the current study was very low (37.8%), which the findings which agrees with similar studies [8,15,24,25] that reported low levels of awareness. Indeed, these findings stand in contrast to a study conducted in Fars Province, Iran, by [26], which suggested that ethnic and cultural background held greater significance than the mothers' level of education in understanding G6PD deficiency risk factors. Notably, awareness of G6PD was highest among central region parents (3.9 times), followed by western region parents (3.1 times) and eastern region parents (2.9 times). No significant awareness levels were reported among parents from the northern and southern regions. This contradicts the findings in Alqahtani T et. al.’s study[8], which stated that the northern region had the highest knowledge level at 52%, while the western regions had lower knowledge levels at 21.4%.

A plausible explanation might be the differences in the sample size and the duration of the studies.

Parental knowledge of G6PD risk factors, with an adjusted odds ratio (adjOR) of 2.5, suggests that those aware are 2.5 times more likely to have a child with G6PD deficiency, highlighting an increased likelihood of G6PD deficiency in their children. It is essential to acknowledge that the significant finding was restricted to parents from the Western region. In the northern region, parents associating family history with G6PD were 75% less likely to have a child with G6PD deficiency, indicating that this belief reduces the likelihood of G6PD deficiency in their children. This finding aligns with the results reported in Algahtani et al.'s [8] study, which linked family history with G6PD deficiency anemia. This discovery highlights how parental viewpoints and convictions impact the occurrence of G6PD deficiency, emphasizing the necessity for precise education and heightened awareness concerning this condition. The overall adjOR of 1.89 indicates a connection between perceiving specific medications as triggers for G6PD deficiency anemia and an increased likelihood of having a G6PD-deficient child, aligning with findings in studies [8,13,15,27,28]. However, this significance was observed solely in Western region parents. Those believing in medication-triggered G6PD are more likely to have a G6PD-deficient child, highlighting the importance of understanding parental views on medication risks and their impact on G6PD prevalence.

Regarding G6PD deficiency clinical presentation, only loss of appetite, jaundice, and dizziness/light-headedness showed significance. The adjOR of 0.63 suggests parents linking loss of appetite with G6PD deficiency anemia are less likely to have G6PD-deficient children, with no significant regional variation. Notably, the association between loss of appetite and G6PD deficiency anemia was exclusively observed in parents with a bachelor's level of education or higher, mirroring the results in [8], which linked higher education to greater awareness of G6PD deficiency anemia. This connection between loss of appetite and G6PD deficiency anemia reduces the likelihood of having a child with G6PD deficiency, emphasizing the significance of parental beliefs regarding anemia and its association with G6PD deficiency. With an adjOR of 3.01, jaundice shows a connection with G6PD deficiency recognition, notably among highly educated parents in the Western region, aligning with [8,27-30] and emphasizing parental beliefs in G6PD deficiency anemia and jaundice. The adjOR of 0.189 suggests that parents perceiving dizziness and light-headedness as G6PD deficiency anemia symptoms are less likely to have a child with G6PD deficiency. This discovery underscores how parental perceptions and beliefs regarding G6PD deficiency symptoms can influence its prevalence. Similar findings, such as the connection between dizziness, light-headedness, and G6PD deficiency anemia, were reported by Alqahtani T. et al. [8].

Understanding G6PD treatment is crucial for its effective management, and this knowledge, particularly concerning fluid intake and blood transfusion, was found to be statistically significant in relation to having a child with G6PD deficiency. Being aware of the need to increase fluid intake during a G6PD attack is associated with a higher likelihood (adjOR 1.53) of G6PD deficiency in offspring, with particular significance observed in the Western region (adjOR 2.69). Nonetheless, this association remains statistically significant (adjOR 2.44) solely among parents with a secondary or diploma education level. It underscores the impact of parental awareness, comprehension, and education in influencing G6PD deficiency prevalence, as similarly documented by Hamali H. et al. and Seneadza NA et. al. [25,29]. Awareness of the potential need for blood transfusions in individuals with G6PD is linked to a higher likelihood of having a child with G6PD deficiency, with significance observed particularly among parents from the central region (adjOR 1.54), underscoring that possessing this knowledge increases the likelihood of having a G6PD-deficient child.

Parents in Saudi Arabia seek G6PD deficiency information from diverse sources, with online resources showing significant associations with increased G6PD deficiency likelihood. However, no significant regional differences exist. Regionally, only parents from the Central (adjOR 8.12) and Western regions (adjOR 4.73) exhibit significance. Seeking G6PD information from healthcare professionals is linked to a higher G6PD deficiency likelihood in these regions, especially among parents aged 31-40 with secondary education or higher. Participation in G6PD-related educational events significantly associate (adjOR 2.71) with an elevated G6PD deficiency likelihood, primarily among Western region parents. In the present study, online sources (25.7%) and healthcare professionals (14.4%) were reported as information sources, which align with findings in Hamali HA’s study [4], where internet and social media (23.3%) and healthcare professionals (14.3%) were similarly reported.

Limitations: The reliance on the self-administered questionnaire is more prone to subjectivity, as participants may provide socially desirable answers or misunderstand certain questions. In addition, the cross-sectional nature of the study limits the ability to establish causation.

## Conclusions

In conclusion, the overall awareness of G6PD deficiency is relatively low; regional disparities exist, with the Central region having the highest awareness. Parental recognition of G6PD risk factors, medication triggers, and clinical symptoms significantly impacts the likelihood of having a G6PD-deficient child. The importance of tailored educational initiatives for specific regions, like the Western region, cannot be overstated. Understanding G6PD treatment, fluid intake, and blood transfusion plays a crucial role, and awareness in these areas is linked to a higher likelihood of having a child with G6PD deficiency. Online resources and healthcare professionals are essential sources of information, particularly in the Central and Western regions. In summary, enhancing parental education, awareness, and access to reliable information are vital steps in addressing G6PD deficiency and improving outcomes for affected children. 

Recommendations

G6PD deficiency should be considered in patients who experience acute hemolysis after exposure to known oxidative medications, infection, or ingestion of fava beans. A diagnosis of G6PD deficiency is most often made through enzymatic activity detection, but molecular analysis may be required in females heterozygous for the disorder. When clinically feasible, rasburicase, primaquine, dapsone, pegloticase, and methylene blue should not be used until a G6PD diagnostic test has been performed. Some of the latest publications should be added. Increasing the awareness of healthcare practitioners, policymakers, and educators regarding this important health problem is needed.
